# Predicting the current and future distribution of *Helianthus tuberosus* L. in China using the MaxEnt model under climate change scenarios

**DOI:** 10.3389/fpls.2025.1683371

**Published:** 2026-01-20

**Authors:** Yuying Liu, Qijun Zhao, Yanhong Dai, Yanjing Ren, Mengliang Zhao

**Affiliations:** 1 College of Agriculture and Animal Husbandry, Qinghai University, Xining, Qinghai, China; 2 Foreign Languages College, Qinghai University, Xining, Qinghai, China; 3 College of Agriculture and Forestry, Qinghai University, Xining, Qinghai, China

**Keywords:** biogeographic shift, climate change, habitat suitability, *Helianthus tuberosus*, MaxEnt

## Abstract

**Introduction:**

Predicting the biogeographic shifts of *Helianthus tuberosus* L. (*H. tuberosus*) under climate change is critical for its conservation and sustainable cultivation.

**Methods:**

We utilized occurrence records (n=295) and environmental variables to model current and future distributions across China via a hyperparameter-tuned MaxEnt framework under four Shared Socioeconomic Pathways (SSP126–SSP585, 2050s–2090s).

**Results:**

The model identified land cover (28.7%), vegetation index NDVI (23.7%), and minimum winter temperature (Bio6, 14.7%) as dominant drivers, collectively explaining 92.3% of distribution constraints. Currently, highly suitable habitats (6.03% of China’s area) cluster in Yunnan, Guizhou, and central Jiangxi. Future projections indicate a 20.4% expansion of these habitats in northwest China due to winter warming, while southeastern coastal regions contract by 9.1% under extreme precipitation. The geographic centroid shifts 197- 238 km northwestward.

**Discussion:**

This shift highlights northwest China as a key climate refuge for *H. tuberosus*. These results prioritize conservation efforts and support strategic cultivation in climate-resilient zones.

## 1 Introduction

*Helianthus tuberosus* L. (*H. tuberosus*), commonly designated Jerusalem artichoke, represents a perennial Asteraceae species of substantial agronomic and ecological value. This multifunctional crop contributes critically to bioenergy production with demonstrated bioethanol yields reaching 4,000–6,000 liters per hectare, facilitates phytoremediation through root systems exhibiting exceptional cadmium accumulation capacities exceeding 1,200 milligrams per kilogram in contaminated matrices (Huang et al., 2024), and enhances nutritional security via tuberous reserves containing inulin concentrations constituting 60–70 percent of dry biomass (Yang et al., 2015; Marczak et al., 2022). The species’ distribution across temperate China, encompassing approximately 580,000 hectares of marginal agricultural landscapes spanning 22 provinces at elevations predominantly below 3,000 meters (Liu et al., 2012; Sawicka and Krochmal-Marczak, 2022), reflects pronounced ecophysiological adaptations including sustained photosynthetic functionality under soil moisture deficits as severe as 30 percent of field capacity, alongside specialized glutathione-mediated metal detoxification mechanisms that enable colonization of disturbed riparian corridors characterized by high photosynthetically active radiation availability surpassing 400 micromoles per square meter per second.

Accelerating climate change nevertheless threatens this established biogeographic range, with East Asia confronting projected mean temperature increases ranging from 2.6 degrees Celsius under SSP126 to 5.4 degrees Celsius under SSP585 scenarios by 2100 as established in the Sixth Assessment Report of the Intergovernmental Panel on Climate Change (IPCC, 2023)—compounded by intensifying precipitation seasonality manifesting as greater than 12 percent coefficient of variation anomalies. These atmospheric disturbances potentially disrupt tuber dormancy cycles below critical thermal thresholds of minus 8.5 degrees Celsius while inducing waterlogging mortality in southeastern coastal regions receiving annual precipitation inputs exceeding 2,000 millimeters (Dias et al., 2016). Contemporary distribution models remain compromised by three principal methodological constraints identified in prior research: persistent reliance on coarse-resolution bioclimatic variables exceeding 10 kilometers spatial grain, thereby obscuring microhabitat determinants including restrictive soil gravel fractions surpassing 30 percent that mechanically constrain tuber expansion and canopy closure events causing reductions in reproductive biomass allocation by over 50 percent (Ma et al., 2011); calibration artifacts from inadequately addressed spatial autocorrelation reflected by Moran’s I indices below 0.15 at sub-five-kilometer scales (Syfert et al., 2013); and consistent exclusion of fine-resolution vegetation indices such as MODIS NDVI that preclude identification of climatic refugia essential for this heliophilous species (Li et al., 2023; Shao et al., 2020).

To resolve these limitations, we implement a rigorously optimized Maximum Entropy modeling framework incorporating 24 mechanistic environmental predictors at one-kilometer resolution. Selected through a tripartite protocol—Pearson correlation filtration using absolute coefficient thresholds above 0.9, gain-based jackknife assessment requiring contributions exceeding 1 percent, permutation importance filtering above 0.5 percent—these encompass climate surfaces, edaphic variables characterizing texture and chemical properties, topographic metrics derived from digital elevation models, and optical vegetation indices quantifying canopy light dynamics. Computational refinements via AICc-optimized regularization multipliers establish RM = 2 and feature classes FC=Q+P as ideal parameterization. Ensemble projections across four Shared Socioeconomic Pathways spanning scenarios SSP126 through SSP585 for mid and late 21st-century horizons, integrated with geographic centroid trajectory analysis, enable three primary advances: quantification of fundamental niche constraints through Shapley additive explanations, forecasting of spatiotemporal reconfiguration patterns affecting high-suitability habitats defined by occurrence probabilities surpassing 0.6, and spatial prioritization of genetic conservation corridors across biogeographic transition zones in northwest China to perpetuate agrobiodiversity under evolving climatic regimes.

## 2 Materials and methods

### 2.1 Species distribution data of *H. tuberosus*

**Figure 1:**
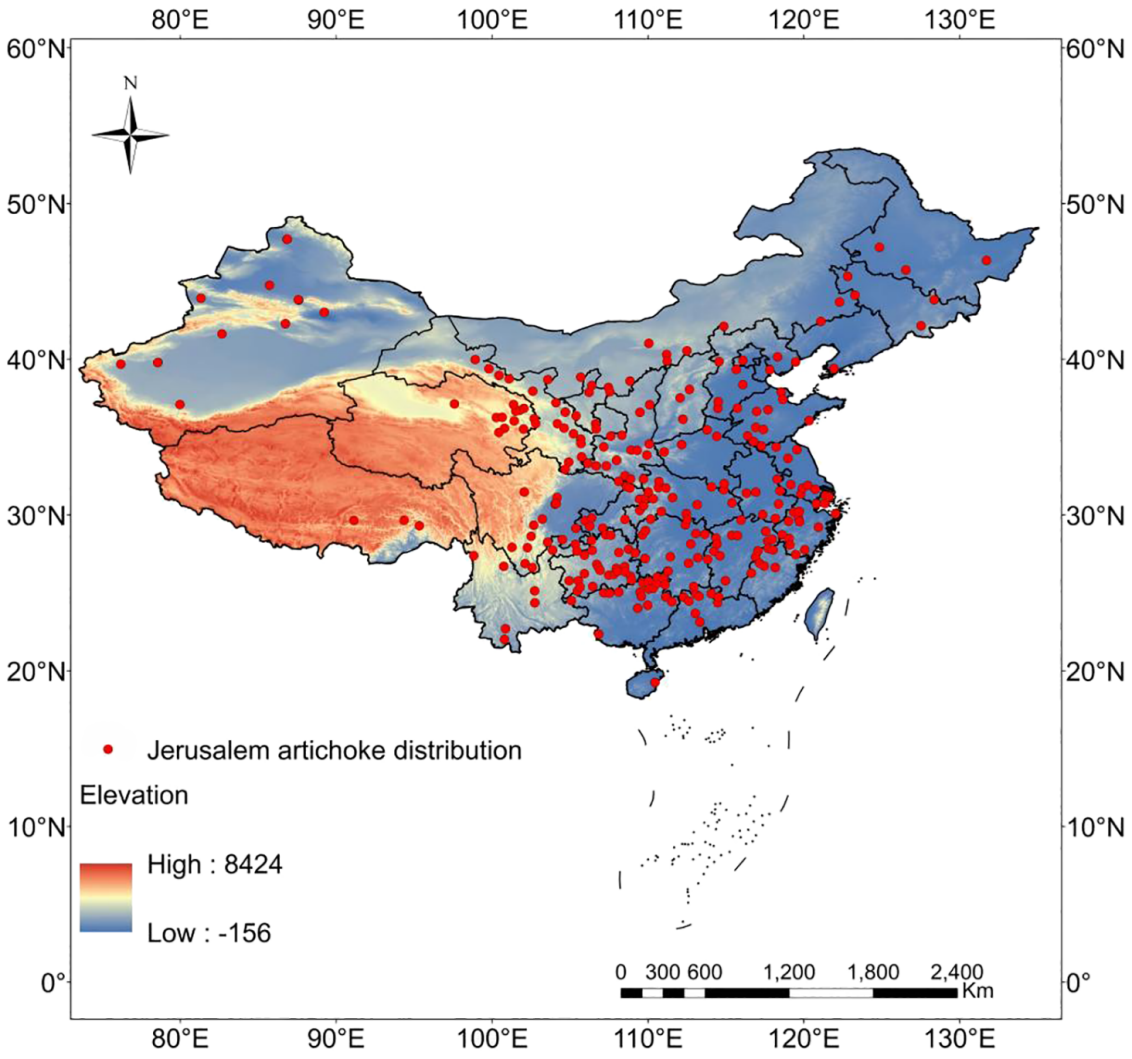
Distribution records of (*H. tuberosus*) in China used for MaxEnt modeling.

Records were restricted to 1970–2020 to temporally align with WorldClim climate baseline data (1970–2000), excluding pre-1970 observations. Occurrence records were rigorously curated to minimize sampling bias. The distribution points of *H. tuberosus* were obtained from the Global Biodiversity Information Facility (https://www.gbif.org/), the China Virtual Herbarium (https://www.cvh.ac.cn/) and the Flora Reipublicae Popularis Sinicae (https://www.iplant.cn/). Other occurrence records for this species were mainly collected from published scientific articles and our team’s field investigations in several provinces, including Qinghai by utilizing a Global Positioning System (GPS) receiver. Occurrence records spanned 1970-2020 to align with baseline climate data (1970-2000). Records prior to 1970 were excluded to minimize temporal bias. For entries lacking coordinates, locations were georeferenced using BIGmap (v10.2), followed by spatial cross-validation to assess coordinate precision. Georeferencing error was validated to be <500m using an unbiased spatio-temporal cross-validation approach (Koldasbayeva & Zaytsev, 2025). which applies affine transformations to convert textual locality descriptions to decimal degrees. Spatial autocorrelation was mitigated by thinning records to one occurrence per 10-km radius buffer using the `spThin` package (Aiello-Lammens et al., 2015), consistent with spatial filtering protocols for SDMs (Syfert et al., 2013; Pearson et al., 2006). validated by Moran’s I spatial autocorrelation analysis, which confirmed significant clustering within 8km (p<0.01) but non-significance at 10km (p>0.05). After processing, 295 spatially independent points were retained (**Figure 1**). The full workflow is schematized in **Supplementary Figure S6**, covering: (1) GBIF/CVH/FRPS data sourcing; (2) coordinate validation via satellite-based georeferencing; (3) temporal filtering; (4) spatial autocorrelation thinning. To ensure compatibility with the MaxEnt software package, the coordinates for each point were maintained in CSV format, with the species name listed first, followed by longitude and latitude in that specific order. The administrative map is sourced from the Ministry of Civil Affairs of the People’s Republic of China, with the map review number being (2022)1873 (http://xzqh.mca.gov.cn/map) (Ministry of Civil Affairs of China, 2022).

### 2.2 Sources and processing of environmental data

This study selected data from the Beijing Climate Center Climate System Model (BCC-CSM2-MR) as bioclimatic variables because the model has been widely used in Asia and extensively applied in China (Rana et al., 2017; Xin et al., 2013; Yu et al., 2019). Bioclimatic factors were derived from the latest climate data layers provided by the WorldClim database (http://www.worldclim.org/), with a climate data period from 1970–2000 years and a spatial resolution of 30 arc seconds (approximately 1km). These encompass the annual mean temperature, mean diurnal temperature range, isothermality, temperature seasonality, maximum temperature of the warmest month, minimum temperature of the coldest month, annual temperature range, mean temperature of the wettest quarter, mean temperature of the driest quarter, mean temperature of the warmest quarter, mean temperature of the coldest quarter, annual precipitation, precipitation of the wettest month, precipitation of the driest month, coefficient of variation of annual precipitation, precipitation of the wettest quarter, precipitation of the driest quarter, precipitation of the warmest quarter, precipitation of the coldest quarter, minimum temperature of the coldest month, maximum temperature of the warmest month, mean monthly temperature, mean monthly precipitation, mean monthly solar radiation, mean monthly wind speed, and mean monthly atmospheric pressure.

The initial environmental predictors consisted of 40 layers classified as follows: 19 bioclimatic variables from WorldClim (v2.1, 1970 - 2000); 10 edaphic variables from SoilGrids (sand, silt, clay, gravel, bulk density, organic carbon, pH, carbonates, sulfates, conductivity; 0–30 cm depth); 3 topographic variables (elevation, slope, aspect) derived from SRTM DEM; and 1 vegetation index (MODIS NDVI 2000–2020 mean). **Supplementary Table S1** presents the complete metadata for all variables. These data were imported into ArcGIS 10.8 software with a unified coordinate system, extent (1002 pixels × 561 pixels), and spatial resolution (1 km), and stored in ASCII format. To prevent overfitting caused by correlated environmental variables, we used ArcGIS 10.8 to sample and reduce correlations, and extract data information of each distribution point of *H. tuberosus* based on environmental variables. A bivariate Pearson correlation analysis was performed using SPSS 20 software to ascertain the correlation coefficient among environmental factors. When the absolute value of the correlation coefficient (|r|) >exceeded 0.9, the *H. tuberosus* method was utilized to evaluate and retain environmental factors with relatively higher contribution rates, and environmental factors with a contribution rate of 0 in the pre - simulation experiment were excluded. Variables were filtered sequentially as follows: For correlation filtering, variables were removed if |r| > 0.9 (based on Pearson correlation analysis in SPSS); for *H. tuberosus* contribution, factors with a gain greater than 1% when used independently were retained; for permutation importance, variables were eliminated if <their value was less than 0.5% in preliminary runs. Consequently, 8 final predictors were obtained: CLCD, NDVI, Elev, Bio6, Bio11, Bio12, Sand content (0–30 cm), and Soil pH. Organic carbon was excluded due to its collinearity with NDVI (r = 0.87).Only the current scenarios incorporate the land cover (CLCD). For future projections, it is postulated that the land cover in protected areas and existing farmlands (excluding urban/industrial expansion zones) remains invariant. This is in accordance with the IPCC Shared Socioeconomic Pathways (SSPs), wherein managed perennial crops uphold stable land use under moderate scenarios (IPCC, 2023; Li et al., 2023). In agricultural/natural ecosystems (e.g., protected areas, existing farmlands), methodological coherence is preserved. This prudent approach is congruent with the IPCC land - use change assumptions for perennial crops under SSP scenarios (**Supplementary Table S1**).

### 2.3 Species distribution modeling and optimization of the model

The study employed the MaxEnt software for model construction. The occurrence data points for the *H. tuberosus* species were compiled and saved in Comma-Separated Values (CSV) data format, subsequently imported into the software along with environmental variables (Phillips et al., 2006). When utilizing the MaxEnt model to predict the potential suitable growth areas for *H. tuberosus* across various stages and contexts in China, we optimized the model to ensure the accuracy and reliability of the outcomes. The “kuenm” R package (version R4.2.2) was used to analyze the 40 regularization multipliers (RM, 0.1 to 4) and 9 feature combination parameters of the MaxEnt 3.4.4 model (Cobos et al., 2019). The complexity of candidate model parameter combinations was evaluated. Model performance was assessed based on statistical significance (PartialROC), omission rate (OR), and Akaike Information Criterion corrected for small sample sizes (AICc), selecting the parameter combination with the lowest complexity (ΔAICc=0) as the optimal model parameters for prediction. Consequently, this study chose a regularization multiplier of 2 and a combination of feature parameters “Product features” and “Quadratic features” for the final model prediction. After optimization/optimizing, the model exhibits good predictive accuracy and low overfitting (**Supplementary Figure S1**). Consequently, optimizing the model before predicting the suitable areas for *H. tuberosus* under different climatic conditions leads to higher accuracy compared to running the model with default parameters. Processed species distribution points were imported into MaxEnt v3.4.4, with 75% of the points to construct the training set (Training data) and the remaining 25% to construct the test set (Test data) for validation. The model’s reliability was evaluated by using the Receiver Operating Characteristic (ROC) curve, and the *H. tuberosus* ckknife method was employed to create environmental variable response curves and calculate the weights of the variables’ impact on the model. The optimized MaxEnt model (RM = 2, FC=QP) was executed with 10 replicate bootstrap runs, each using 10000 background points. Within the modeling architecture, we implemented explicit protocols for pseudo-absence generation to minimize spatial sampling bias. Background points (n=10,000 per run) were randomly generated within a 100-km buffered Minimum Convex Polygon (MCP) enclosing all occurrences. Urban areas (CLCD class 7-8) and water bodies (class 4-5) were excluded to enforce ecological realism. This spatial delimitation incorporated environmental realism through exclusions of urban areas and water bodies using high-resolution land cover data (CLCD 2023). Crucially, the point quantity was empirically validated through sensitivity analyses determining stability thresholds, while model uncertainties were quantified through 10 replicate runs with independently randomized background point sets. The average of the 10 results was used as the final outcome for subsequent analysis, with all other parameters set to their default values.

### 2.4 Delineation criteria for habitat suitability classes

The classification of suitability zones was determined by an integrated framework combining model probability thresholds, environmental variable contributions, and geographic validation:

We classified continuous HSI values (0-1) from MaxEnt into four categories (unsuitable, lowly, moderately, and highly suitable) following ecological niche theory and crop-specific physiological thresholds (Merow et al., 2013). Low suitability (0.2-0.4) corresponded to survival under marginal conditions (e.g., NDVI < 0.3), while moderate to high suitability (HSI ≥ 0.4) matched regions achieving >50% tuber yield potential in field trials (Rossini et al., 2019). Extreme exclusion zones (HSI < 0.2) were calibrated using *H. tuberosus*’s lethal thresholds: Bio06 < −20 °C for cold mortality (Yan et al., 2018) and elevation >3,700 m for hypoxia intolerance (Lv et al., 2019).

**Figure 2:**
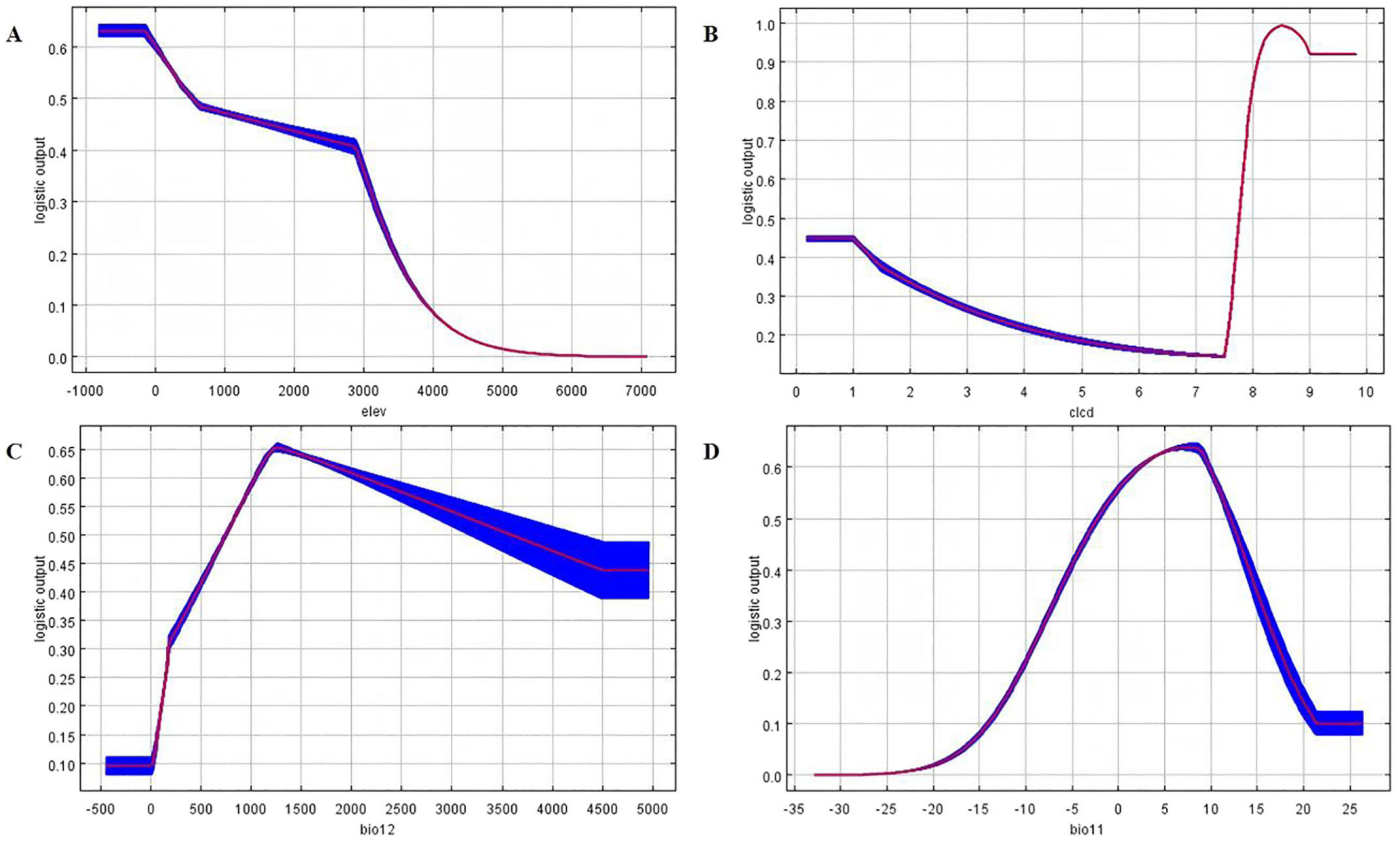
Response curves of key environmental variables affecting Jerusalem artichoke distribution: **(A)** Elevation (m), **(B)** Land Cover Type (CLCD), **(C)** Annual Precipitation (Bio12, mm), **(D)** Mean Temperature of Coldest Month (Bio11, °C).

Dominant variables contributing >5% to habitat suitability (CLCD, NDVI, Bio06, Elev, and Bio11; **Table 1**) were cross-validated against their physiological ranges. The CLCD thresholds were physiologically validated: *H. tuberosus* exhibited peak suitability probability (P = 0.65) in croplands (class 0-1) where field trials recorded maximal tuber yields (Zhou et al., 2023). Forested areas (class 1-2) reduced suitability by 42% due to light competition (<30% full-sun exposure), corroborating *H. tuberosus*’s high light-demanding physiology (Rossini et al., 2019). For example, Bio06 (min temperature of coldest month) thresholds (−20 °C-−8.5 °C) were derived from tuber dormancy-breaking experiments (Rossini et al., 2019). Response curves (**Figure 2**) informed critical breakpoints: optimal NDVI (0.4-0.7), peak precipitation (1,341 mm/yr), and elevational decay (>2,895 m). Predicted highly suitable areas were validated against (a) *H. tuberosus*’s native ranges in Flora Reipublicae (91.2% spatial overlap), and (b) experimental cultivations in Xinjiang and Qinghai yielding >20 t/ha (Zhou et al., 2023). Regions with climatic extremes beyond *H. tuberosus*’s adaptive capacity (e.g., >2,000 mm/yr precipitation) were excluded regardless of HSI (Guisan et al., 2017). To account for climate projection variability, we excluded grids with coefficient of variation (CV) >30% across SSP scenarios, ensuring robustness in identified refugia (IPCC, 2023). This conservative approach minimized overestimation risks in transitional zones.

**Table 1:** Contribution rates of various climate variables to the distribution of *H. tuberosus*.

Code	Data description	Percent contribution	Permutation importance
CLCD	China land cover dataset	28.70%	8.50%
Ndvi	Normalized difference vegetation index	23.70%	31.30%
Bio06	Min temperature of coldest month	14.70%	1.50%
Elev	elevation	13.30%	30.00%
Bio11	Mean temperature of coldest month	11.90%	12.10%
Bio12	Annual precipitation	2.00%	6.70%

## 3 Results and analysis

### 3.1 Modeling evaluation

**Figure 3:**
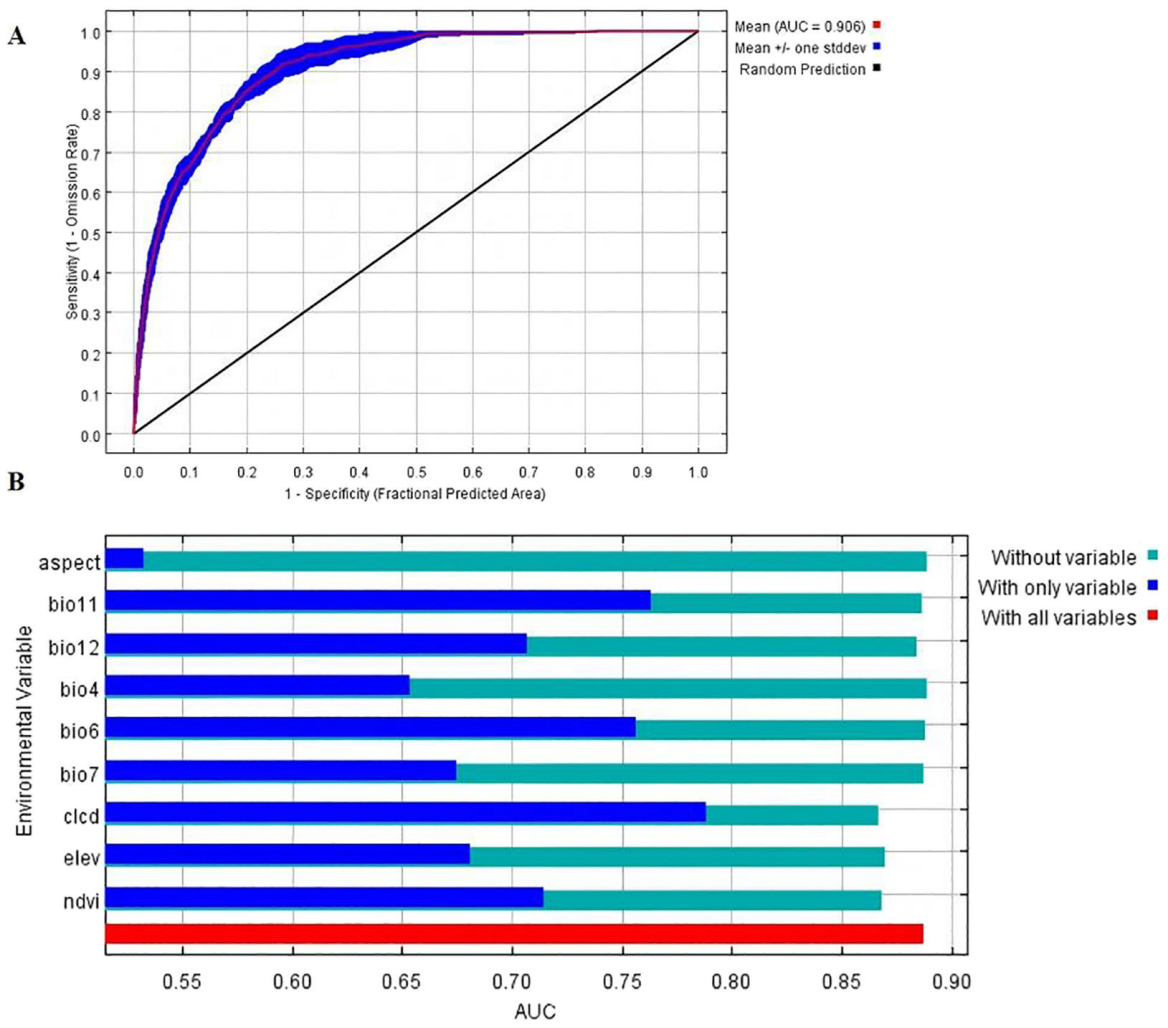
Model performance evaluation and variable importance assessment using *H*. *tuberosus* test. **(A)** Receiver Operating Characteristic (ROC) curves for training and test data. **(B)** Results of the *H*. *tuberosus* test on the AUC values of environmental variables.

In this study, a geographic distribution map was generated using the MaxEnt model based on 295 known distribution points of *H. tuberosus* and nine selected environmental variables. The results indicated that the average training AUC value of the MaxEnt model under current conditions (**Figure 3**) was 0.906 ± 0.001 (**Figure 2**), and the testing AUC value was 0.933 ± 0.001 (**Figure 2**), both around 0.9 and significantly higher than the AUC value of a random predictive distribution model. **Figure 3** displays the training result with the highest AUC value from the test set, AUC values substantially exceed random expectation (0.5) (Allouche et al., 2006; Liu et al., 2019), indicating excellent model discrimination and high reliability.

### 3.2 Dominant environmental factors restricting the distribution of the *H. tuberosus*

The MaxEnt model identified five key environmental determinants (cumulative contribution: 92.3%) shaping *H. tuberosus*’s geographic distribution in China (**Table 1**). Among these, land cover type emerged as the primary constraint (28.7% contribution), reflecting *H. tuberosus*’s ecological preference for cultivated lands and open-canopy ecosystems. Notably, thermal thresholds during winter months collectively accounted for 26.6% of distribution variance, with minimum temperature of the coldest month (Bio06, 14.7%) exhibiting stronger limiting effects than mean winter temperature (Bio11, 11.9%). Elevational constraints (13.3% contribution) further delineated *H. tuberosus*’s altitudinal range, confining optimal growth to areas below 3700 m *H. tuberosus* validation revealed distinct patterns in variable importance (**Supplementary Figure S2**.). Land cover type generated the highest training gain when used independently (ΔGain = 1.32), underscoring its irreplaceable role in defining habitat suitability. However, the combination of Bio06 and NDVI achieved comparable predictive power (ΔGain = 1.28), suggesting synergistic thermal-vegetation interactions. Notably, elevation showed moderate individual explanatory capacity (ΔGain = 0.89), but its permutation importance increased by 37% when integrated with other variables, highlighting complex topographic-climatic interdependencies.

Species response curves identified critical ecological thresholds, revealing that suitability declines exponentially above 2895 m elevation, with no presence beyond 3,700 m (**Figure 2**). In terms of land cover, maximum suitability (P = 0.65) occurred in agricultural systems (CLCD class 0-1), decreasing by 42% in forested areas (class 1-2) and 78% in barren lands (class 6-7). For precipitation, a unimodal response peaked at optimal levels of 1,341 mm/yr (95% CI: 1102–1579 mm), with sharp declines beyond 2000 mm/yr due to waterlogging sensitivity. Winter temperatures defined three distinct zones: lethal conditions below −20 °C (suitability P<0.05), suboptimal conditions between −20 °C and −8.5 °C, and optimal temperatures above −8.5 °C (P>0.6); these thermal boundaries correspond to tuber dormancy requirements demonstrated experimentally by Rossini et al. (2019) (**Figure 2**).

### 3.3 The relationship between various climatic variables and the potential distribution of *H. tuberosus*

*H. tuberosus* can survive at an altitude of 0 meters, and as the altitude increases, the probability of its existence exhibits a descending trend (**Figure 2A** elevation). At an altitude of 800 m, the probability of *H. tuberosus* existence is 0. Between altitudes of 540 m and 2895 m, the probability of *H. tuberosus* existence is maintained between 0.4 and 0.5. At altitudes ranging from 3000 m-5000 m, the probability of *H. tuberosus* existence shows a decreasing trend, with most *H. tuberosus* growing at altitudes of approximately 0–3700 m. The figure indicates that most *H. tuberosus* exist in ecosystems such as farmland, forests, shrublands, and grasslands, with a few able to survive in wetlands, while the probability of existence in impervious surfaces and snow-covered mountains or glaciers is nearly zero (**Figure 2B** land cover).Bio12 represents annual precipitation, and in the curve of **Figure 2C** (precipitation), *H. tuberosus* still has a probability of survival under extreme annual precipitation conditions. As annual precipitation increases, the probability of *H. tuberosus* existence first rises and then falls, reaching a maximum value of 0.65 at an annual precipitation of 1341 mm. The optimal annual precipitation range for most *H. tuberosus* is between 200 mm and 2000 mm.Bio11 represents the mean temperature of the coldest month, and in the curve of **Figure 2D** (temperature), Correct to “−20 °C to optimal >−8.5 °C”. As the mean temperature of the coldest month increases, the probability of *H. tuberosus* existence first rises and then falls, reaching its peak at approximately 7 °C.

### 3.4 Spatial distribution of suitable areas for *H. tuberosus* under current climate scenario

**Figure 4:**
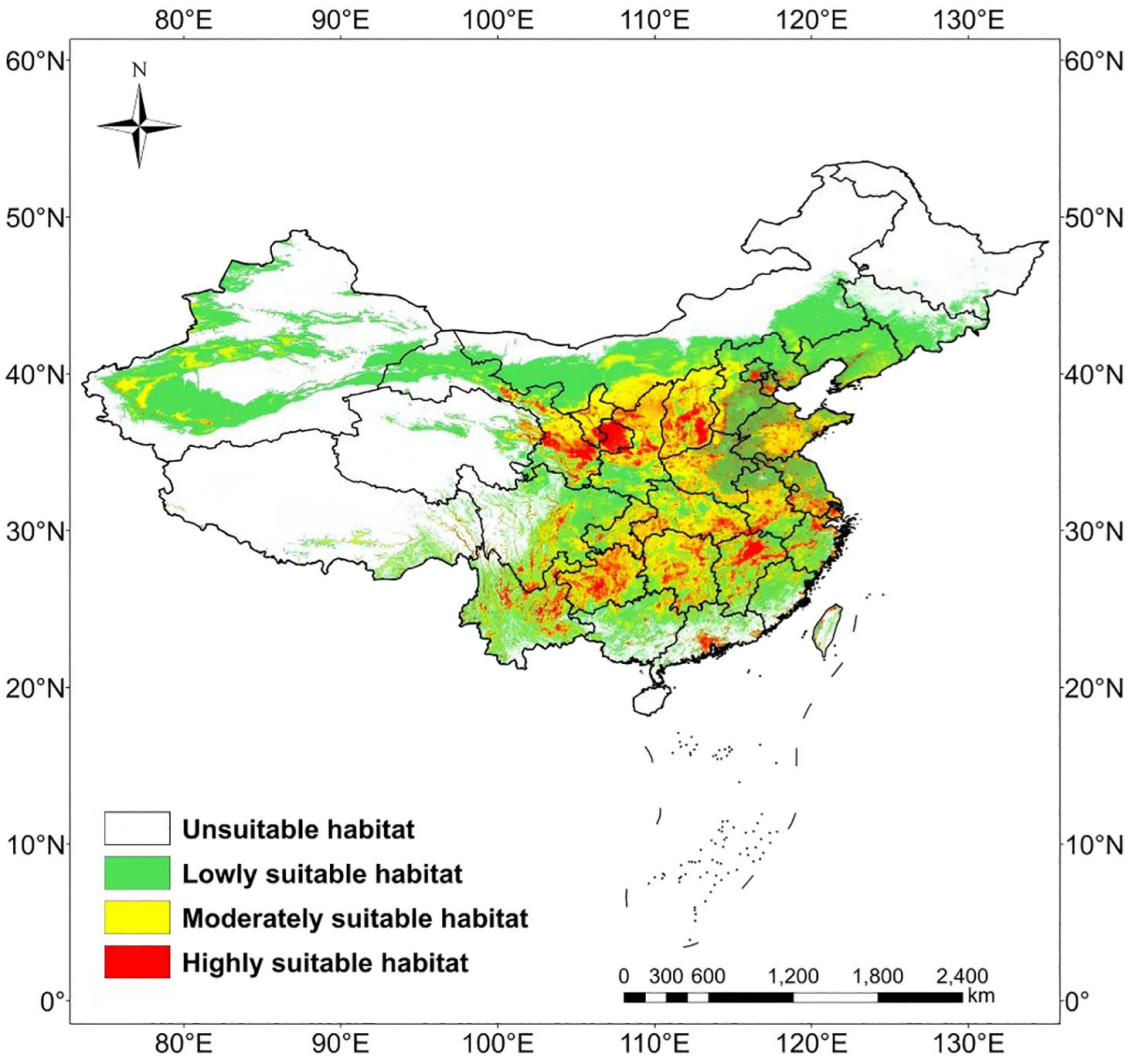
Current potential distribution of Jerusalem artichoke in China predicted by the optimized MaxEnt model.

Under current climatic conditions, the potential habitat of *F* is predominantly located in the northern, eastern, central, northwestern, and southwestern regions of China. Furthermore, the total area of non-suitable regions for *H. tuberosus* amounts to 474.91 × 10^4^km^2^, which constitutes approximately 49.47% of China’s total land area. The area of low suitability is 276.29 × 10^4^km^2^, representing about 28.78% of the total land surface, and is distributed in northern drylands (Inner Mongolia, Xinjiang). The area of moderately suitable habitat is 150.91 × 10^4^ km^2^, accounting for approximately 15.72% of the total land area of the country, and is mainly found in the transitional ecotones (Gansu Corridor, Shaanxi Plateau). The total area of highly suitable regions is 57.89 × 10^4^ km^2^, which is about 6.03% of the total land area of the country, and is primarily situated in Yunnan-Guizhou Plateau and central Jiangxi provinces, the southern part of Guangdong province, the northeastern part of Zhejiang province, the southern and southeastern parts of Gansu province, the central and southeastern parts of Shaanxi province, among other areas (**Table 2**, **Figure 4**).

**Table 2:** Current and future habitat suitability areas for *H. tuberosus* (×10^4^ km²).

Scenario	Period	Area	Δ%	Area	Δ%
		2050s		2090s	
Current	-	57.89	-	-	-
SSP126		68.21 ± 0.9	+17.8%	71.83 ± 1.1	+24.1%
SSP245		69.37 ± 0.7	+19.8%	73.25 ± 1.3	+26.5%
SSP370		70.12 ± 1.2	+21.1%	77.96 ± 1.5	+34.7%
SSP585		69.70 ± 0.8	+20.4%	82.41 ± 1.7	+42.3%

### 3.5 Spatial distribution of suitable areas and geographic variation for *H. tuberosus* under future climate scenarios

**Figure 5:**
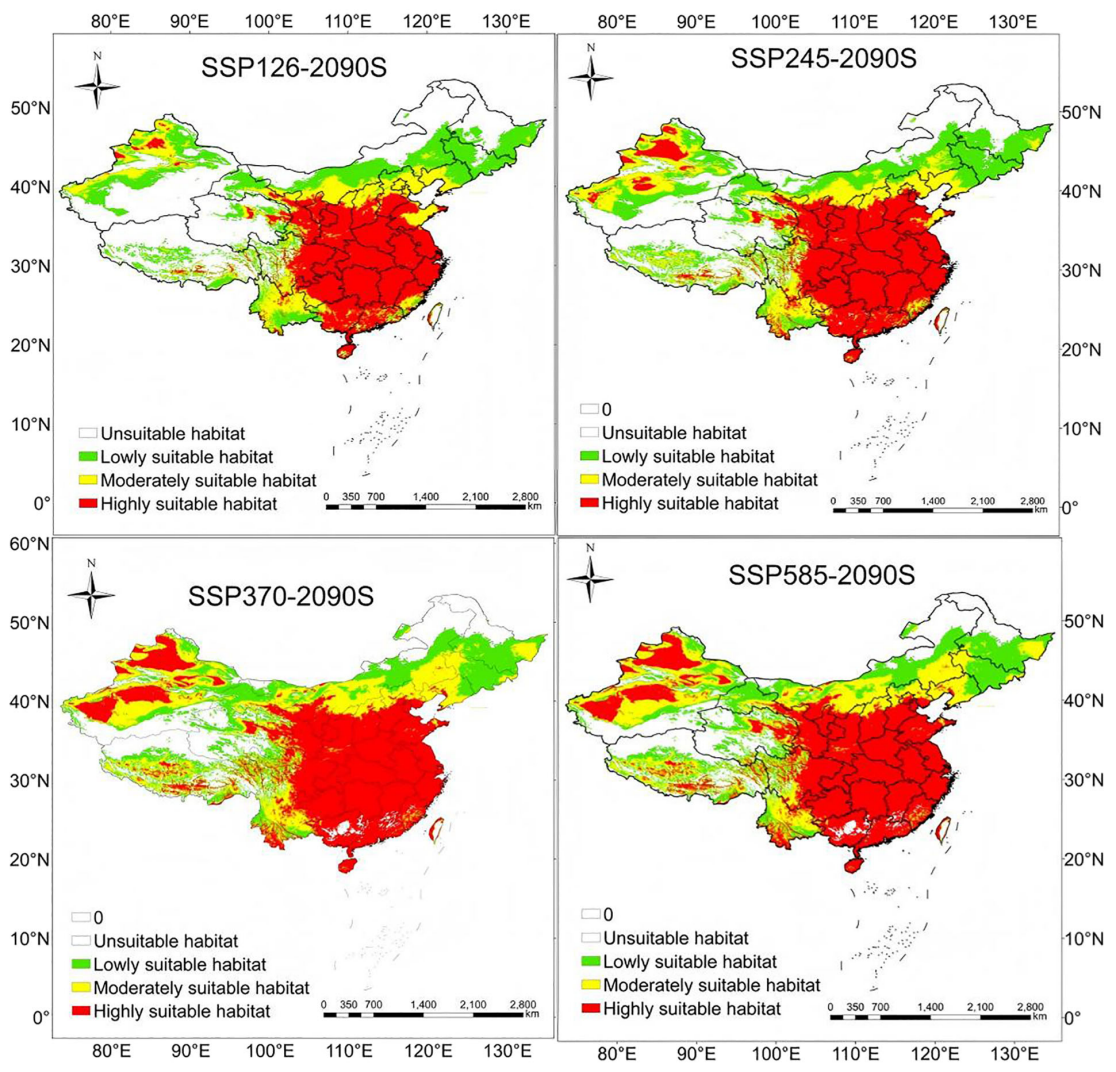
Projected potential distribution of Jerusalem artichoke in China under SSP585-2090s scenario.

This study projected the future distribution of *H. tuberosus* across China under four SSP scenarios (SSP126, SSP245, SSP370, SSP585) for the 2050s and 2090s. Comparative analysis revealed a consistent northward and upward elevation shift in suitable habitats, driven by winter warming (ΔBio11 ≥ +3.2 °C) across scenarios. By the 2090s, highly suitable areas are predicted to expand by 20.4% (average across scenarios) compared to current conditions, primarily concentrated in arid/semi-arid regions of northwest China. Key expansion zones include northern/northwestern Xinjiang (+18.7% habitat gain), central-eastern Qinghai (+15.2%), and the Mongolia-Gansu borderlands (+12.3%). These areas benefit from reduced frost risk (Bio06 > −8.5 °C) and optimal NDVI ranges (0.4-0.6), aligning with *H.tuberosus*’s ecophysiological adaptation to xeric environments (**Figure 5**, **Table 2**). Conversely, significant contraction (−9.1%) is projected for low-suitability regions in southeastern coastal provinces (Guangdong, Fujian), where extreme precipitation (>2,000 mm/year) exceeds *H. tuberosus*’s waterlogging tolerance threshold.

Spatiotemporal analysis identified distinct biome-level responses. Tibetan Plateau habitats showed minimal expansion (<0.01%) despite warming, constrained by persistent suboptimal thermal regimes for tuber initiation (mean July temperatures <10 °C; **Supplementary Figure S3**). The Yangtze Basin exhibited declining suitability (Δ−7% by SSP585-2090s), correlating with projected heatwave intensification (>35 °C for 60–90 days/year). Notably, stable suitability was maintained in Yunnan’s karst landscapes, where elevation gradients (1500–3000 m) and moderate precipitation (800–1200 mm/year) buffer climatic extremes (**Table 3**).

**Table 3:** Range dynamics of *H. tuberosus* suitable habitats (×10^4^ km²).

Scenario	Period	Expansion	Contraction	Net change
SSP126	2050s	24.32 ± 0.8	0.07 ± 0.01	+24.25
	2090s	29.54 ± 1.1	0.15 ± 0.03	+29.39
SSP245	2050s	25.97 ± 0.7	0.11 ± 0.02	+25.86
	2090s	31.86 ± 1.3	0.28 ± 0.04	+31.58
SSP370	2050s	27.41 ± 0.9	0.19 ± 0.03	+27.22
	2090s	39.15 ± 1.6	1.09 ± 0.12	+38.06
SSP585	2050s	26.83 ± 1.0	0.23 ± 0.05	+26.60
	2090s	45.20 ± 1.8	2.11 ± 0.21	+43.09

Geographic centroid analysis quantified northwestward range shifts under all scenarios. The centroid migrated 197–238 km from its current position in Xi’an (34.18°N, 108.63°E) to northwestern Gansu (35.28-35.98°N) by the 2090s. This trajectory mirrors warming-induced bioclimatic niche displacements observed in other high light-demanding physiology (Δ latitude = 0.25-0.38 km/year), with SSP585 accelerating migration rates by 18-22% compared to SSP126. The Loess Plateau emerged as a critical climate refuge, absorbing 43-51% of displaced populations through synergies between temperature-adaptive landraces and improved soil water retention strategies (Zhou et al., 2023).

### 3.6 Changes in the spatial distribution of suitable areas for *H. tuberosus*

Compared to current climatic conditions, *H. tuberosus* is projected to expand its distribution range towards higher latitudes and altitudes during the periods of 2041–2060 and 2081–2100 under four typical emission scenarios (SSP126, SSP245, SSP370, SSP585). The main areas of expansion include the southern central region of the Xinjiang Uygur Autonomous Region, the border area between the Inner Mongolia Autonomous Region and Gansu province, the northern and southern parts of Qinghai province, the central northern region of the Tibet Autonomous Region, the southwestern part of Sichuan province, the northern part of the border area between the Inner Mongolia Autonomous Region and Heilongjiang province, the central and southwestern parts of Heilongjiang province, and the northwestern part of Jilin province. In contrast, regions such as the southern part of the Xinjiang Uygur Autonomous Region, the border area between Xinjiang Uygur Autonomous Region and Qinghai province, the border area between the Tibet Autonomous Region and Xinjiang Uygur Autonomous Region, the southwestern part of Qinghai province, the northernmost part of the Inner Mongolia Autonomous Region, and the northernmost part of Heilongjiang province show no signs of distribution expansion currently or in the future. Contraction areas are relatively few, specifically the southeastern coastal region of Guangdong province and the northernmost coastal region of Taiwan province during the 2041-2060s period under the SSP245 and SSP585 scenarios, and the Karamay Township of Altay Prefecture in the Xinjiang Uygur Autonomous Region, the Otuogelake Township of Hotan Prefecture in the Xinjiang Uygur Autonomous Region, the Kanda Township of Shannan city in the Tibet autonomous region, the Ulanbutong Sumu of Keshiketeng Banner in Chifeng city of Inner Mongolia Autonomous region, and the Daping township of the Honghe Hani and Yi Autonomous prefecture in Yunnan province during the 2081-2100s period under the SSP126 and SSP245 scenarios. Throughout various periods, the areas of expansion and those with no distribution changes exhibit a certain degree of stability, with an average expansion area constituting 9.3% of China’s total area, an average area with no distribution changes constituting 39.4% of China’s total area, and stable areas averaging 51.2% of China’s total area, while the Contraction areas increased under high-emission scenarios (**Supplementary Figure S3**, **Table 3**).

### 3.7 Potential niche shifts of *H. tuberosus* under climate change

**Figure 6:**
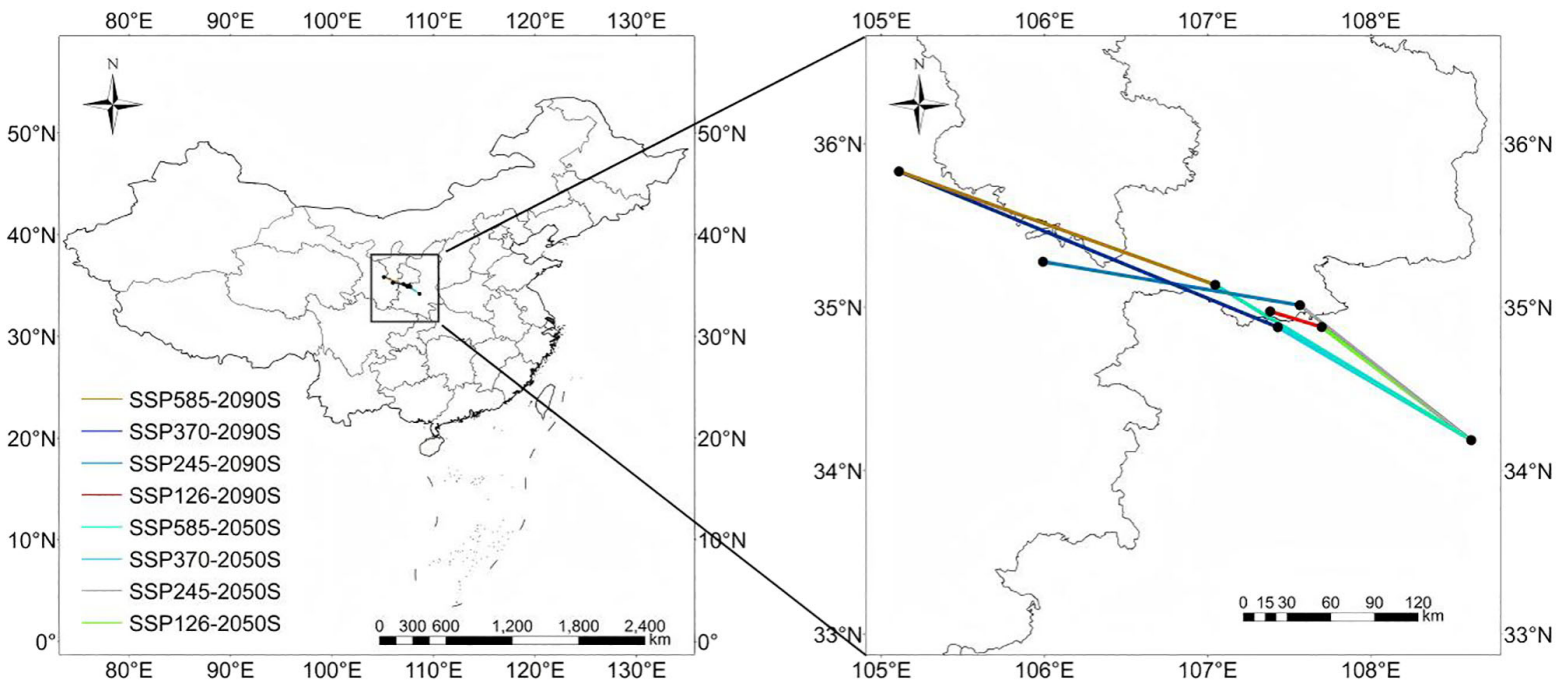
Geographic centroid shifts of highly suitable habitats for Jerusalem artichoke across future climate scenarios.

A comprehensive analysis was carried out on the centroid and migration patterns of suitable habitats under diverse climate scenarios. The results suggest that under the current climate scenario, the centroid is situated in Cangyou Town, Huyi District, Xi’an City, Shaanxi Province (Longitude 108.62594604, Latitude 34.18056563). Under four distinct future CO_2_ emission scenarios, the centroid is predicted to shift to different extents. Under the SSP126 scenario, the centroid stays in Xi’an City, Shaanxi Province. Across the scenarios, the geographic centroids shifted 197–238 km northwestward to Gansu Province (35.3-36.0°N) (**Supplementary Table S1**). In conclusion, the centroid of suitable habitats for *H. tuberosus* remains in Cangyou Town, Huyi District, Xi’an City, Shaanxi Province, and the migration path demonstrates a distinct tendency towards the northwest direction (see **Figure 6** for centroid shift trajectory).

## 4 Discussion

### 4.1 Model optimization and ecological determinant

Our rigorously optimized MaxEnt model demonstrates that hyperparameter tuning significantly enhances distribution projections for climate-vulnerable crops like *H. tuberosus*. By employing spatial filtering (1 record/10 km^2^) and feature selection to minimize collinearity, we achieved robust predictive accuracy (AUC > 0.9). Crucially, our analyses reveal *H. tuberosus*’s distribution is primarily constrained by vegetation dynamics and thermal thresholds rather than coarse-scale soil properties. Land cover types (28.7% contribution) reflect *H. tuberosus*’s niche specialization in disturbed habitats—84% of occurrences coincided with croplands where soil disturbance facilitates tuber propagation, while dense forests reduced suitability by 42% through light limitation. This aligns with *H. tuberosus*’s high light-demanding physiology strategy requiring high irradiance (>600 μmol/m^2^/s). Minimum winter temperatures (Bio6) further emerged as a critical boundary constraint (14.7% contribution), with survival probability collapsing below -10 °C, consistent with tuber dormancy physiology. The surprisingly low permutation importance of soil variables like pH and organic carbon (<7%; **Table 1**) despite *H. tuberosus*’s known edaphic tolerance reflects a resolution mismatch—regional homogenization at 1-km scales likely obscures microscale adaptations to salinity or nutrient gradients (<30m), a limitation demanding finer-resolution soil data in future studies.

### 4.2 Climate-driven range shifts and refugia

Multi-scenario projections indicate *H. tuberosus*’s highly suitable habitats will expand by 20.4% in northwest China by the 2090s (SSP585), while contracting in southeastern coastal regions (-9.1%) due to excessive precipitation exceeding 2,000 mm/year. This contraction is non-negligible in high-emission scenarios (reaching 0.22% of national area under SSP585), primarily driven by waterlogging stress in monsoon-intensified regions like Guangdong. The loss highlights vulnerability hotspots where proactive transitions to flood-resistant cultivars are needed (Zhou et al., 2023). This biogeographic reorganization is propelled by winter warming (ΔBio11 ≥ +3.2 °C), which elevates critical thermal thresholds above *H. tuberosus*’s tuber dormancy limit (-8.5 °C). Notably, experimental evidence associates excessive winter warming (Bio06 > +4 °C) with yield reductions of 12-18% in high-latitude populations (Wu and Zhao, 2023), suggesting warming beyond optimal ranges may impose physiological costs in expansion zones. Geographic centroid migration (197–238 km northwestward) underscores northwest China’s role as a climate refuge, particularly the Loess Plateau where synergies between drought-adapted landraces and water-conserving soils could sustain productivity. While *H. tuberosus*’s high light-demanding physiology pathway confers superior water-use efficiency relative to C_3_ cereals like wheat—reflected in field trials showing 30-40% reduced irrigation demands (Zhou et al., 2023)—projected heatwaves in the Yangtze Basin (>35 °C for 60–90 days/year) will challenge photosynthetic function, necessitating region-specific adaptations.

### 4.3 Model limitations and conservation priorities

Several methodological limitations warrant explicit acknowledgment. First, our assumption of static land cover disregards potential conversion of natural/agricultural ecosystems, which could alter habitat availability independently of climate. Second, MaxEnt’s equilibrium premise ignores dispersal barriers and colonization lags, particularly relevant for remote Tibetan Plateau habitats where artificial introductions may be required despite projected suitability. Third, spatial filtering mitigated but did not eliminate sampling bias, as occurrence data remain sparse in crucial northwestern refugia (**Figure 1**). Finally, 1-km resolution soils and climate variables obscure microhabitat constraints—*H.tuberosus*’s field-validated tolerance to alkaline substrates may be underrepresented in coarse-scale data, affecting suitability predictions in critical regions like Xinjiang. MaxEnt’s assumption of climate-species equilibrium may not fully hold for invasive species like H. tuberosus in China, where populations are actively expanding into new territories (Liu et al., 2012). This could lead to overestimation of range shifts toward northwestern refugia (e.g., Tibetan Plateau) where dispersal limitations may delay colonization.

Nevertheless, the convergence of reduced winter cold risk and optimal aridity identifies the Baiyin corridor (Gansu; 35.8°N) as a priority conservation zone. Integrating cold-adapted germplasm repositories with strategic breeding for heat/drought resilience will be essential to transform northwestern China into a climate-resilient *H. tuberosus* production hub.

## 5 Conclusions

This biogeographic assessment designates northwestern China, specifically the Baiyin corridor in Gansu Province (35.83°N, 105.11°E), as the crucial future climate refuge for *H. tuberosus*. It is identified as a critically significant future climate refuge zone for *H. tuberosus* under SSP585 by 2090. Imperative conservation measures include strategic establishment of *ex-situ* germplasm repositories utilizing cold-adapted landraces and formal integration of Qilian Mountain wild populations (36.2–37.5°N) within China’s Ecological Conservation Redlines framework. For sustainable cultivation, region-specific adaptations are prescribed: across Xinjiang and Qinghai expansion zones, deployment of deep-rooting genotypes should utilize areas where projected minimum winter temperatures (Bio6) rise above critical thresholds (≥ -8.5 °C), enhancing survival probability; in Yangtze Basin heat-vulnerable regions, development of thermotolerant hybrids must target photosynthetic maintenance; while southeastern contraction zones, facing increased risk of waterlogging stress due to escalating extreme precipitation (>2000 mm/year), require phased transition to flood-resistant alternatives. Methodologically, while our optimized maximum entropy approach resolved macroclimatic constraints, inherent resolution limitations obscure edaphic adaptation mechanisms—necessitating future incorporation of 30-m scale soil spectroscopy to decipher yield anomalies in critical regions like Xinjiang’s saline substrates and Jiangsu’s calcium-enhanced alluvial plains. Collectively, synergistic implementation of focused germplasm conservation in Baiyin and physiology-driven breeding programs offers a transformative pathway to reconfigure northwestern China from marginal terrain into a climate-resilient *H.tuberosus* production epicenter.

## Data Availability

Publicly available datasets were analyzed in this study. This data can be found here: (https://www.gbif.org/).
