# Supplementary material for: Predicting the current and future distribution of Helianthus tuberosus L. in China using the MaxEnt model under climate change scenarios
Source: Front Plant Sci. 2026 Jan 20;16:1683371. doi: 10.3389/fpls.2025.1683371 (PMC12864416; doi:10.3389/fpls.2025.1683371)

Supplementary Material

# Supplementary Figures and Tables

## Supplementary Figures

**Supplementary Table 1.** Full List of 40 Initial Environmental Variables. All variables used prior to correlation and Jackknife filtering.

| **Category** | **Variable Code** | **Variable Name/Description** | **Units** | **Depth/Temporal** |
| --- | --- | --- | --- | --- |
| **Bioclimatic (26)** | Bio01 | Annual Mean Temperature | °C × 10 | 1970-2000 baseline |
|  | Bio02 | Mean Diurnal Range (Mean of monthly max-min) | °C × 10 |  |
|  | Bio03 | Isothermality (Bio02/Bio07)×100 | % |  |
|  | Bio04 | Temperature Seasonality (SD×100) | - |  |
|  | Bio05 | Max Temperature of Warmest Month | °C × 10 |  |
|  | Bio06 | Min Temperature of Coldest Month | °C × 10 |  |
|  | Bio07 | Temperature Annual Range (Bio05-Bio06) | °C × 10 |  |
|  | Bio08 | Mean Temperature of Wettest Quarter | °C × 10 |  |
|  | Bio09 | Mean Temperature of Driest Quarter | °C × 10 |  |
|  | Bio10 | Mean Temperature of Warmest Quarter | °C × 10 |  |
|  | Bio11 | Mean Temperature of Coldest Quarter | °C × 10 |  |
|  | Bio12 | Annual Precipitation | mm |  |
|  | Bio13 | Precipitation of Wettest Month | mm |  |
|  | Bio14 | Precipitation of Driest Month | mm |  |
|  | Bio15 | Precipitation Seasonality (CV) | % |  |
|  | Bio16 | Precipitation of Wettest Quarter | mm |  |
|  | Bio17 | Precipitation of Driest Quarter | mm |  |
|  | Bio18 | Precipitation of Warmest Quarter | mm |  |
|  | Bio19 | Precipitation of Coldest Quarter | mm |  |
|  | Tmean_01 | January Mean Temperature | °C × 10 |  |
|  | Tmean_02 | February Mean Temperature | °C × 10 |  |
|  | Tmean_03 | March Mean Temperature | °C × 10 |  |
|  | Prec_12 | December Precipitation | mm |  |
|  | Srad_06 | June Solar Radiation | kJ/m²/day |  |
|  | Wind_03 | March Wind Speed | m/s |  |
|  | Pres_08 | August Atmospheric Pressure | kPa |  |
| **Edaphic (10)** | Sand_top | Sand Content (0-5 cm) | % w/w | 0-5 cm depth |
|  | Silt_top | Silt Content (0-5 cm) | % w/w | 0-5 cm depth |
|  | Clay_top | Clay Content (0-5 cm) | % w/w | 0-5 cm depth |
|  | Gravel_top | Gravel Volume Fraction (0-5 cm) | % vol | 0-5 cm depth |
|  | BD_0-30 | Bulk Density (0-30 cm) | kg/m³ | 0-30 cm depth |
|  | OC_0-30 | Organic Carbon (0-30 cm) | g/kg | 0-30 cm depth |
|  | pH_0-30 | Soil pH (H₂O) (0-30 cm) | - | 0-30 cm depth |
|  | Caco3_0-30 | Carbonate Content (0-30 cm) | g/kg | 0-30 cm depth |
|  | Sulfates_15-30 | Sulfate Content (15-30 cm) | mg/kg | 15-30 cm depth |
|  | EC_5-15 | Electrical Conductivity (5-15 cm) | dS/m | 5-15 cm depth |
| **Topographic (3)** | Elev | Elevation | m |  |
|  | Slope | Slope | Degrees |  |
|  | Aspect | Aspect | ° (0-360) | N=0°, E=90°, S=180°, W=270° |
| **Vegetation (1)** | NDVI | Normalized Difference Vegetation Index | -1 to +1 | 2000-2020 mean |

**Supplementary Table S2.**MaxEnt Model Parameter Optimization Results

| **RM** | **Feature Combination** | **AICc Value** | **Omission Rate** | **Partial ROC (p-value)** | **Selected** |
| --- | --- | --- | --- | --- | --- |
| **0.5** | **LQHPT** | **152.3** | **0.12** | **0.08** |  |
| **1.0** | **QP** | **142.1** | **0.09** | **0.03*** |  |
| **2.0** | **QP** | **136.8** | **0.04** | **0.01*** | **✓** |
| **3.0** | **L** | **148.7** | **0.15** | **0.12** |  |
| **4.0** | **H** | **155.2** | **0.18** | **0.10** |  |

**Supplementary Table S3.**Geographic centroids of highly suitable habitats for JA across climate scenarios

| **Scenario** | **Period** | **Longitude (°E)** | **Latitude (°N)** | **Administrative Location** |
| --- | --- | --- | --- | --- |
| **Current** | **-** | **108.6259** | **34.1806** | **Cangyou Town, Hu County, Shaanxi** |
| **SSP126** | **2050s** | **107.7031** | **34.8949** | **Zhangba Town, Linyou County, Shaanxi** |
| **SSP126** | **2090s** | **106.8792** | **35.4518** | **Yaojiagou Village, Fengxiang District, Shaanxi** |
| **SSP245** | **2050s** | **107.0924** | **35.1826** | **Shuangmiao Village, Qianyang County, Shaanxi** |
| **SSP245** | **2090s** | **106.3247** | **35.6985** | **Maji Town, Zhangjiachuan County, Gansu** |
| **SSP370** | **2050s** | **106.8543** | **35.3761** | **Donghua Town, Huating County, Gansu** |
| **SSP370** | **2090s** | **105.6789** | **36.0124** | **Hanji Town, Huining County, Gansu** |
| **SSP585** | **2050s** | **106.4728** | **35.2947** | **Wenfeng Town, Longxi County, Gansu** |
| **SSP585** | **2090s** | **105.1032** | **35.8375** | **Hanjiaji Town, Tongwei County, Gansu** |

**Supplementary Figure S1.**MaxEnt model parameter optimization
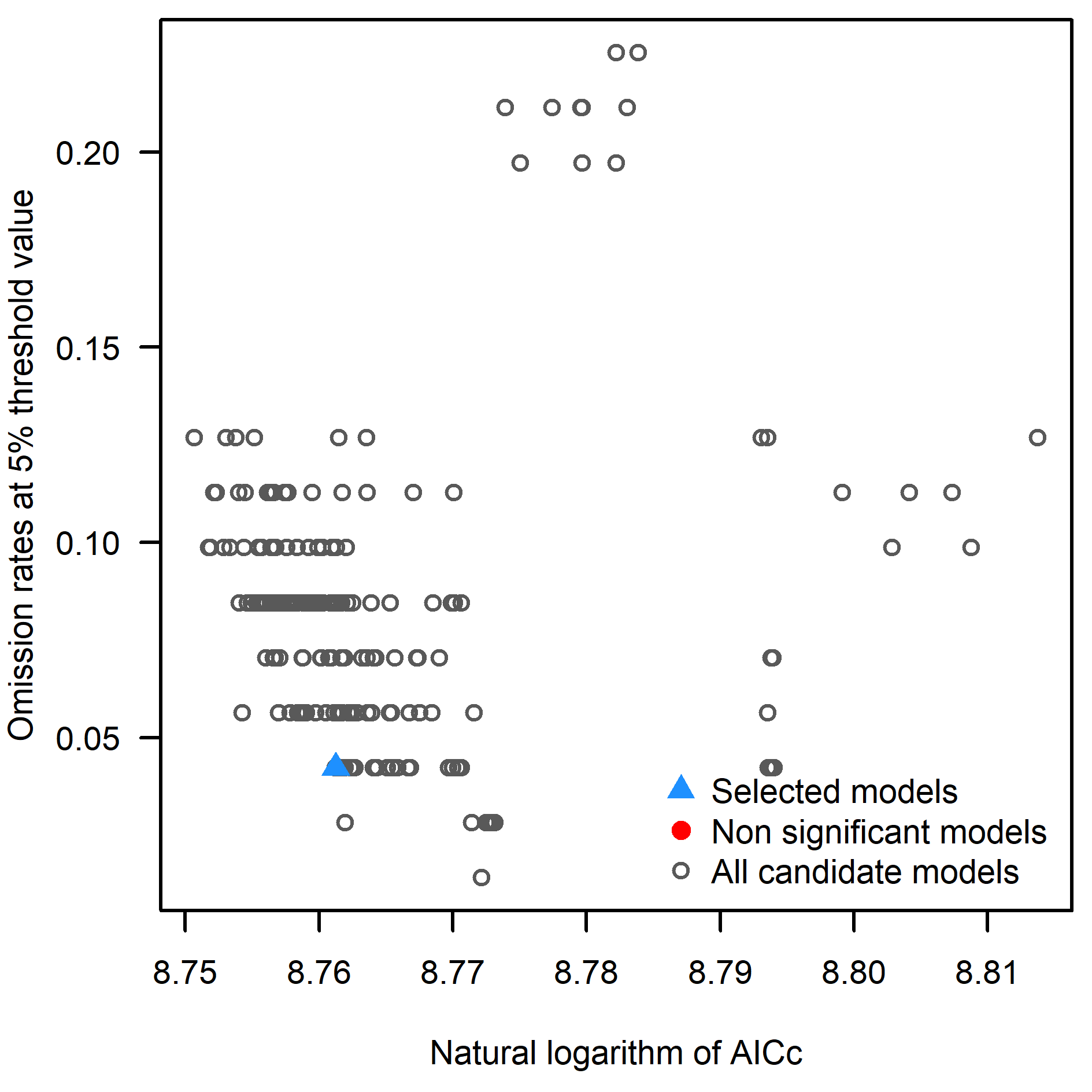
 results.


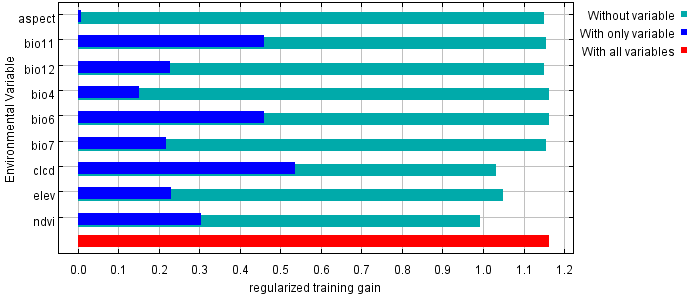
**Supplementary Figure S2.** Jackknife test of variable training gain for Jerusalem artichoke distribution.

**Supplementary Figure S3.**Habitat change dynamics (expansion, stable, contraction) for Jerusalem artichoke under different future climate scenarios.


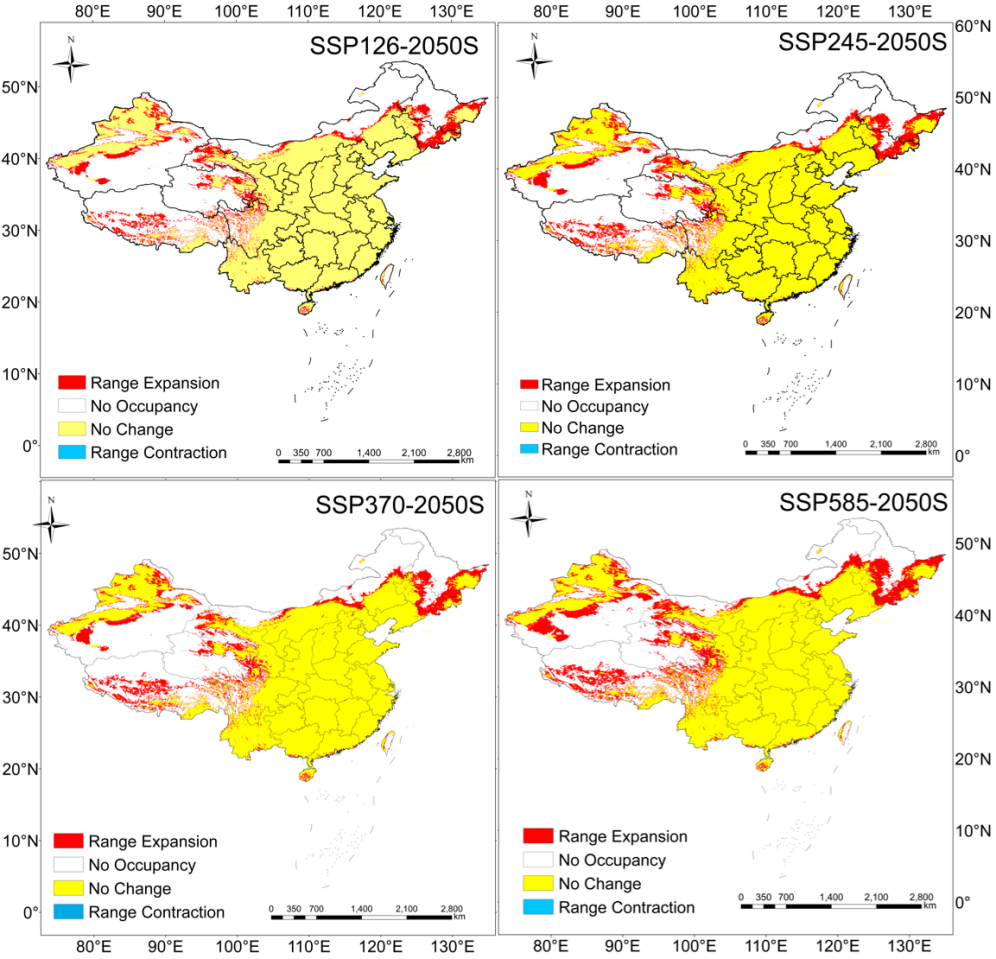


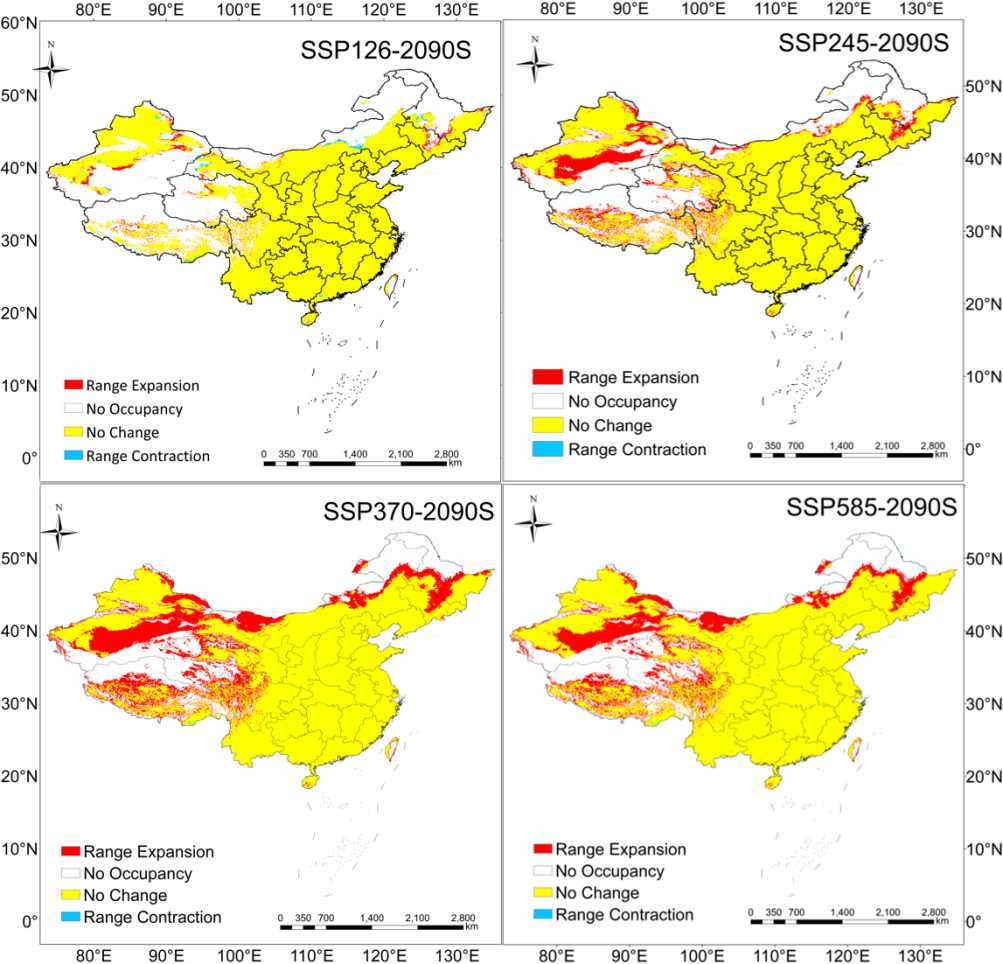


**Supplementary Figure S6.**Data Cleaning Flowchart :


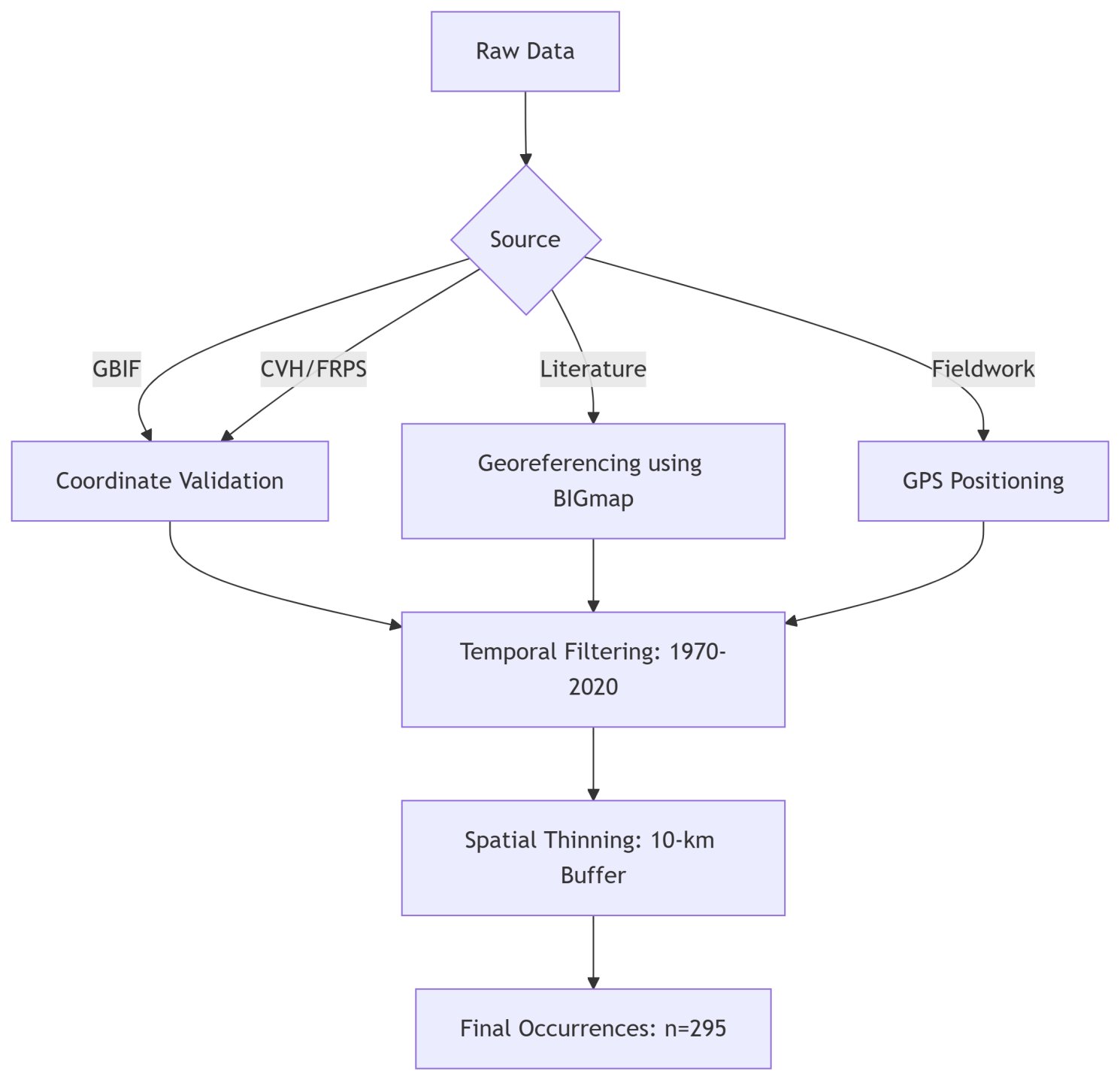

Supplement: Supplementary file 1 [file Table1.docx]
